# Molecular Evidence of *Bartonella melophagi* in Ticks in Border Areas of Xinjiang, China

**DOI:** 10.3389/fvets.2021.675457

**Published:** 2021-06-22

**Authors:** Jun Ni, Qiaoyun Ren, Hanliang Lin, Malike Aizezi, Jin Luo, Yi Luo, Zhan Ma, Ze Chen, Wenge Liu, Junhui Guo, Zhiqiang Qu, Xiaofeng Xu, Zegong Wu, Yangchun Tan, Jinming Wang, Youquan Li, Guiquan Guan, Jianxun Luo, Hong Yin, Guangyuan Liu

**Affiliations:** ^1^State Key Laboratory of Veterinary Etiological Biology, Key Laboratory of Veterinary Parasitology of Gansu Province, Lanzhou Veterinary Research Institute, Chinese Academy of Agricultural Sciences, Lanzhou, China; ^2^Animal Health Supervision Institute of Xinjiang, Ürümqi, China; ^3^Jiangsu Co-innovation Center for the Prevention and Control of Important Animal Infectious Disease and Zoonose, Yangzhou University, Yangzhou, China

**Keywords:** *Bartonella melophagi*, ticks, ixodidae, cattle, sheep, Xinjiang

## Abstract

*Bartonella* are gram-negative intracellular bacteria; certain species of *Bartonella* can cause diseases in mammals and humans. Ticks play a major role in the transmission of *Bartonella*. Xinjiang is the largest province in China according to land area and has one-third of the tick species in China; the infection rate of *Bartonella* in ticks in the Xinjiang border areas has not been studied in detail. Therefore, this study investigated tick infections by *Bartonella* in Xinjiang border areas, and the purpose of the study was to fill in gaps in information regarding the genetic diversity of tick infections by *Bartonella* in Xinjiang. We tested 1,549 tick samples from domestic animals (sheep and cattle) for *Bartonella* using *ribC*-PCR. Positive samples from the *ribC*-PCR assay for *Bartonella* spp. were further subjected to PCR assays targeting the ITS, *rpoB* and *gltA* genes followed by phylogenetic analyses. *Bartonella* DNA was detected in 2.19% (34/1,549) of tick samples, and the ITS, *rpoB* and *gltA* genes of *ribC* gene-positive samples were amplified to identify nine samples of *Bartonella melophagi*. In this study, molecular analysis was used to assess the presence and genetic diversity of *B. melophagi* in ticks collected from sheep and cattle from Xinjiang, China. This study provides new information on the presence and identity of *B. melophagi* in ticks from sheep and cattle.

## Introduction

Bacteria of the genus *Bartonella* are obligate gram-negative intracellular bacteria that primarily infect mammalian erythrocytes, macrophages, monocytes, and endothelial and dendritic cells (1, 2). *B. bacilliformis* was the only named species of *Bartonella* before 1990. At present, more than 36 species have been discovered, 17 of which are related to human and animal diseases (3).

Ruminants have played a vital role in ecology, agriculture and the economy worldwide. They are widely used by humans for different purposes, such as ecological indicators, dairy products and meat. At present, there are 5 kinds of *Bartonella* related to ruminants: *B. bovis, B. chomelii, B. schoenbuchensis, B. capreoli*, and *B. melophagi*. An increasing number of Candidatus *Bartonella* species and different genotypes have been reported in ruminants (4–7). Previous studies have shown that *Bartonella* DNA is amplified in ruminant-related blood-eating arthropods, including Hippoboscidae flies from Europe and Algeria (8, 9), *Stomoxys* spp. and *Haematobia* spp. from California (10), *Rhipicephalus microplus* from Brazilian Cerrado and Taiwan (11, 12), *Haematopinus tuberculatus* from Brazilian Cerrado and *H. quadripertusus* from Israel (12, 13), *Haemaphysalis bispinosa* from Malaysia (14), *H. flava* and *Ixodes persulcatus* from Korean (15), *H. longicornis* from North Korea and Korean (15, 16), *Hyalomma anatolicum* from Pakistan (17), *Ctenocephalides felis* from Tunisia (18), sheep keds (*Melophagus ovinus*) from Central Europe and northeastern Algeria (19, 20), and deer keds (*Lipoptena cervi*) from Norway (6).

*Bartonella* uses humans, cats, dogs, mice, horses, cows, rabbits and other wild animals around the world as hosts (3, 21, 22). It can be transmitted by insect vectors, such as ticks, human body louse, cat fleas, and sand flies (3, 22, 23). Ticks are a widely distributed vector insect worldwide and can transmit and carry a variety of zoonotic pathogens, including viruses, bacteria, parasites, spirochetes and *Rickettsia* (24, 25).

As the largest province in China, Xinjiang has a vast territory, a diverse ecological environment, and rich species. At present, Xinjiang has one-third of the tick species found in China (26). Ticks in Xinjiang have been shown to transmit multiple pathogenic infections, such as *Babesia, Anaplasma, Rickettsia, Theileria* and *Tularemia* (27–30); however, there are only a few studies on *Bartonella* infections in ticks in Xinjiang. Previous studies confirmed that sheep keds (*M. ovinus*) in Xinjiang were infected with *B. melophagi* (31). Therefore, we aimed to investigate *Bartonella* infection in ticks collected from the border areas of Xinjiang and its genetic diversity to fill in the existing lacunae.

## Materials and Methods

### Specimen Collection and Morphological Identification of the Ticks

**Figure 1:**
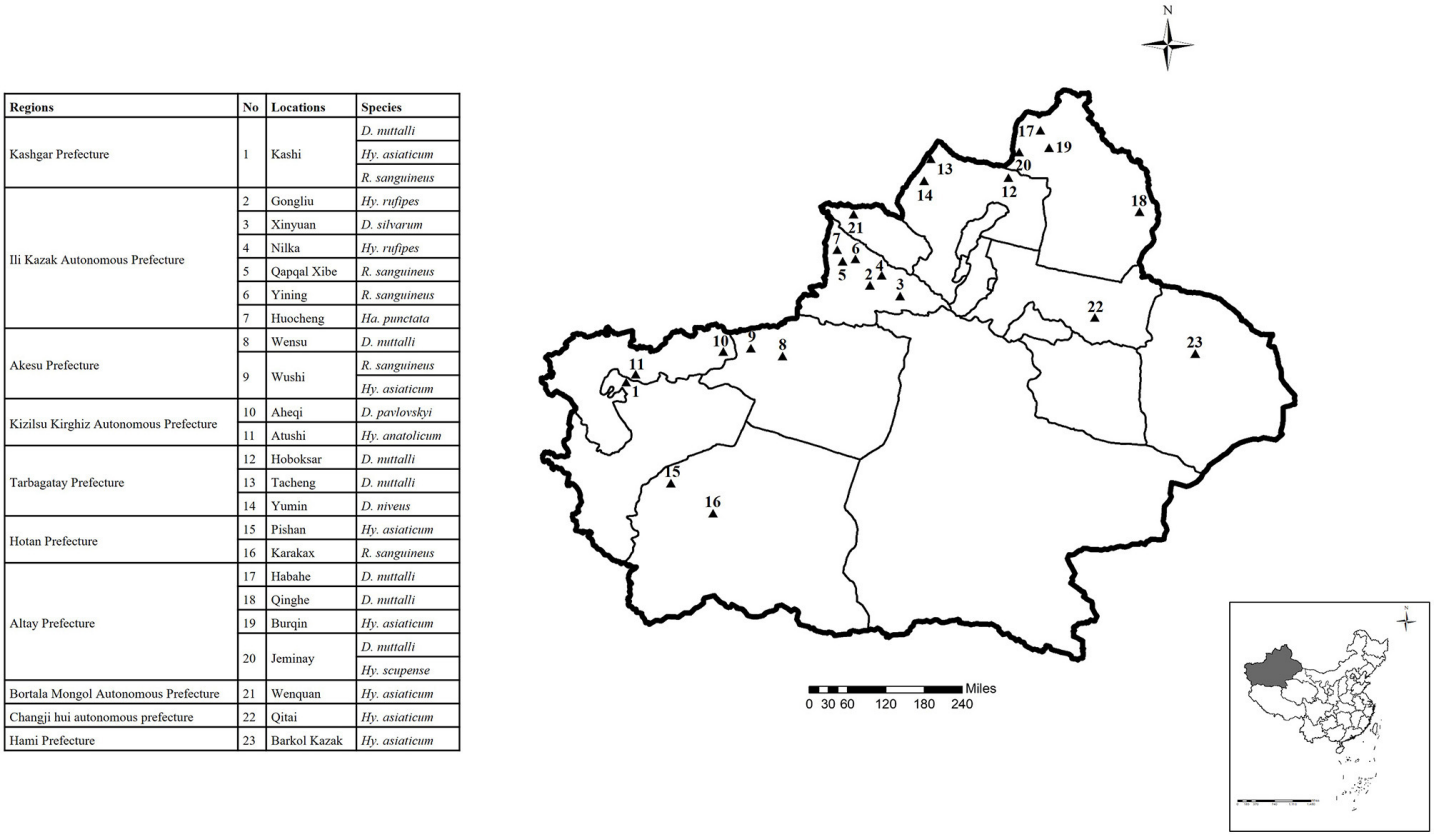
Locations of the sample sites for tick collection in the border areas of Xinjiang (different locations are coded by number; 1-23 indicate the sampling points). The map was made by ArcMap 10.2 (https://developers.arcgis.com/).

From 2018 to 2019, we randomly collected tick samples from cattle and sheep at 23 sampling sites in 10 regions on the Xinjiang border, China (Figure 1, Table 1). Sampling sites included Kashi, Gongliu, Xinyuan, Nilka, Qapqal, Yining, Huocheng, Wensu, Wushi, Aheqi, Atushi, Hoboksar, Tacheng, Yumin, Pishan, Karakax, Habahe, Qinghe, Burqin, Jeminay, Wenquan, Qitai, and Barkol Kazak. The collected samples were stored in 50 mL centrifuge tubes and delivered to the laboratory. The ticks were identified based on morphological criteria following the descriptions provided by Deng et al. (32). Tick samples were collected with permission from the farmers.

**Table 1:** *Bartonella* spp. detection in ticks collected from different localities of the Xinjiang border.

**Regions**	**Locations**	**Species**	**Host animals**	**No. positive/No. examined**
Kashgar prefecture	Kashi	*D. nuttalli*	Sheep	3/120
		*Hy. asiaticum*	Sheep	10/40
		*R. sanguineus*	Sheep	0/70
Ili Kazak autonomous prefecture	Gongliu	*Hy. rufipes*	Cattle	0/78
	Xinyuan	*D. silvarum*	Sheep	0/80
	Nilka	*Hy. rufipes*	Cattle	0/12
	Qapqal Xibe	*R. sanguineus*	Sheep	0/100
	Yining	*R. sanguineus*	Sheep	0/90
	Huocheng	*Ha. punctata*	Cattle	0/92
Akesu prefecture	Wensu	*D. nuttalli*	Sheep	0/88
	Wushi	*R. sanguineus*	Sheep	1/48
		*Hy. asiaticum*	Cattle	4/30
Kizilsu Kirghiz autonomous prefecture	Aheqi	*D. pavlovskyi*	sheep	7/146
	Atushi	*Hy. anatolicum*	Cattle	0/25
Tarbagatay prefecture	Hoboksar	*D. nuttalli*	Sheep	0/23
	Tacheng	*D. nuttalli*	Cattle	0/10
	Yumin	*D. niveus*	Cattle	0/32
Hotan prefecture	Pishan	*Hy. asiaticum*	Sheep	0/30
	Karakax	*R. sanguineus*	Sheep	1/30
Altay prefecture	Habahe	*D. nuttalli*	Cattle	0/48
	Qinghe	*D. nuttalli*	Sheep	0/46
	Burqin	*Hy. asiaticum*	Cattle	0/48
	Jeminay	*D. nuttalli*	Sheep	0/15
		*Hy. scupense*	Cattle	0/2
Bortala mongol autonomous prefecture	Wenquan	*Hy. asiaticum*	Cattle	0/60
Changji hui autonomous prefecture	Qitai	*Hy. asiaticum*	Cattle	8/138
Hami prefecture	Barkol Kazak	*Hy. asiaticum*	Cattle	0/48
	Total			34/1,549

### DNA Extraction and Molecular Analysis

Tick samples were placed in 50 mL sterilized centrifuge tubes and washed individually twice with 75% ethanol followed by rinsing with double-distilled water (ddH_2_O) until the liquid was clear. DNA was extracted from each sample using a QIAamp DNA mini kit (Qiagen, Hilden, Germany) according to the manufacturer's protocol, and the extracted DNA was stored at −20°C. Initial screening of *Bartonella* DNA was performed using *ribC*-PCR. To further characterize the positive samples in this study, positive samples by *ribC*-PCR assay for *Bartonella* spp. were further subjected to PCR assays targeting the ITS, *rpoB* and *gltA* genes, followed by phylogenetic analyses (33–36). A negative control was prepared with ddH_2_O; a positive control was prepared in our laboratory using DNA extracted from *Hy. asiaticum* infected by *B. melophagi*. The PCR products of the partial *gltA, rpoB, ribC* and ITS genes were purified using an E.Z.N.A.^®^ gel extraction kit (Omega, USA) and cloned into the pGEM-T Easy vector (Promega, USA).

### Sequencing and Phylogenetic Analysis

The nucleotide sequences were confirmed by bidirectional sequencing at TSINGKE Biotech, China. The nucleotide sequences were compared with the reference sequences of GenBank (www.ncbi.nlm.nih.gov/nuccore/) by SeqMan (www.dnastar.com/); the forward and reverse primers of the sequences after bidirectional sequencing were selected using SeqMan; extra sequences at the ends of the forward and reverse primers were removed, and corrected calibration was performed after manual adjustment as needed. The sequences were aligned with sequences downloaded in GenBank using MAFFT (37). ModelFinder (38) was used to select the best model using the BIC criterion. Phylogenetic inference was founded on maximum likelihood (ML) analysis through the IQ-TREE (39) program of PhyloSuite V1.2.2 (40). The trees were edited in Figtree v1.4.3 (https://github.com/rambaut/figtree/releases). The confidence values for each branch of the phylogenetic tree were determined by using 1,000 repeat analyses.

## Results

A total of 1,549 tick samples were collected from livestock in various regions of the Xinjiang border (Table 1). All tick samples represented a single family [Ixodidae], four genera [*Dermacentor* (*n* = 608), *Hyalomma* (*n* = 511), *Rhipicephalus* (*n* = 338), and *Haemaphysalis* (*n* = 92)], and ten species [*D. nuttalli* (*n* = 350), *D. pavlovskyi* (*n* = 146), *D. silvarum* (*n* = 80), *D. niveus* (*n* = 32), *Hy. rufipes* (*n* = 90), *Hy. scupense* (*n* = 2), *Hy. anatolicum* (*n* = 25), *Hy. asiaticum* (*n* = 394), *R. sanguineus* (*n* = 338), and *Ha. punctata* (*n* = 92)] (Table 2).

**Table 2:** The positive rate of *Bartonella* spp. in ticks was detected by *ribC*-PCR.

**Family**	**Genus**	**Species**	**No. positive/No. examined**	**Infection rate (%)**	**Animal host**
Ixodidae	*Dermacentor*	*D. nuttalli*	3/350	0.86	3 from sheep, cattle
		*D. pavlovskyi*	7/146	4.79	Sheep
		*D. silvarum*	0/80	0.00	Sheep
		*D. niveus*	0/32	0.00	Cattle
	*Hyalomma*	*Hy. scupense*	0/2	0.00	Cattle
		*Hy. rufipes*	0/90	0.00	Cattle
		*Hy. asiaticum*	22/394	5.58	10 from sheep, 12 from cattle
		*Hy. anatolicum*	0/25	0.00	Cattle
	*Haemaphysalis*	*Ha.punctata*	0/92	0.00	Cattle
	*Rhipicephalus*	*R. sanguineus*	2/338	0.59	Sheep
	Total		34/1,549	2.19	

In this study, we assayed *Bartonella* in 1,549 tick samples from livestock (cattle and sheep) using *ribC*-PCR and detected *Bartonella* DNA in 2.19% (34/1,549) of tick samples. The positive samples had the following proportions: *D. nuttalli* 3 (*n* = 350; 0.86%), *D. pavlovskyi* 7 (*n* = 146; 4.79%), *Hy. asiaticum* 22 (*n* = 394; 5.58%), and *R. sanguineus* 2 (*n* = 338; 0.59%). Meanwhile, the *gltA*, ITS and *rpoB* genes were amplified by PCR and sequencing. A total of 17 *Bartonella ribC* (three from *D. nuttalli*, seven from *Hy. asiaticum*, five from *D. pavlovskyi*, and two from *R. sanguineus*), nine *gltA* (two from *D. nuttalli*, four from *Hy. asiaticum*, two from *D. pavlovskyi*, and one from *R. sanguineus*), 12 ITS (three from *D. nuttalli*, six from *Hy. asiaticum*, two from *D. pavlovskyi*, and one from *R. sanguineus*) and eight *rpoB* (one from *D. nuttalli*, four from *Hy. asiaticum*, two from *D. pavlovskyi* and one from *R. sanguineus*) sequences were obtained.

The results of sequence analysis showed that there were no basic differences among the *rpoB* (*n* = 8), *gltA* (*n* = 9), *ribC* (*n* = 17), and ITS (*n* = 12) sequences obtained in this study. The sequence identity between the examined *B. melophagi* and other known *Bartonella* species was 84–100% for *rpoB*, 76.20–99.80% for *ribC*, 83.10–100% for *gltA*, and 68.90–96.40% for ITS. The sequence identity between the examined *B. melophagi* and known *B. melophagi* was 100% for *rpoB* (EF605288.1), 99.80% for *ribC* (99.80), 99.50–100% for *gltA* (AY724768.1, MG701233.1, and AY724769.1), and 96.40% for ITS (JF834886.1) (Table 3).

**Table 3:** Identity of *rpoB, gltA, ribC* and ITS sequences with *Bartonella* spp. and *B. melophagi*.

	**Sequence identity (%)**	
**Gene**	***Bartonella* spp**.	***B. melophagi***
*rpoB*	84.00–100	100 (EF605288.1)
*ribC*	76.20–99.80	99.80 (EF605287.1)
*gltA*	83.10–100	99.50–100 (AY724768.1, MG701233.1, and AY724769.1)
ITS	68.90–96.40	96.40 (JF834886.1)

**Figure 2:**
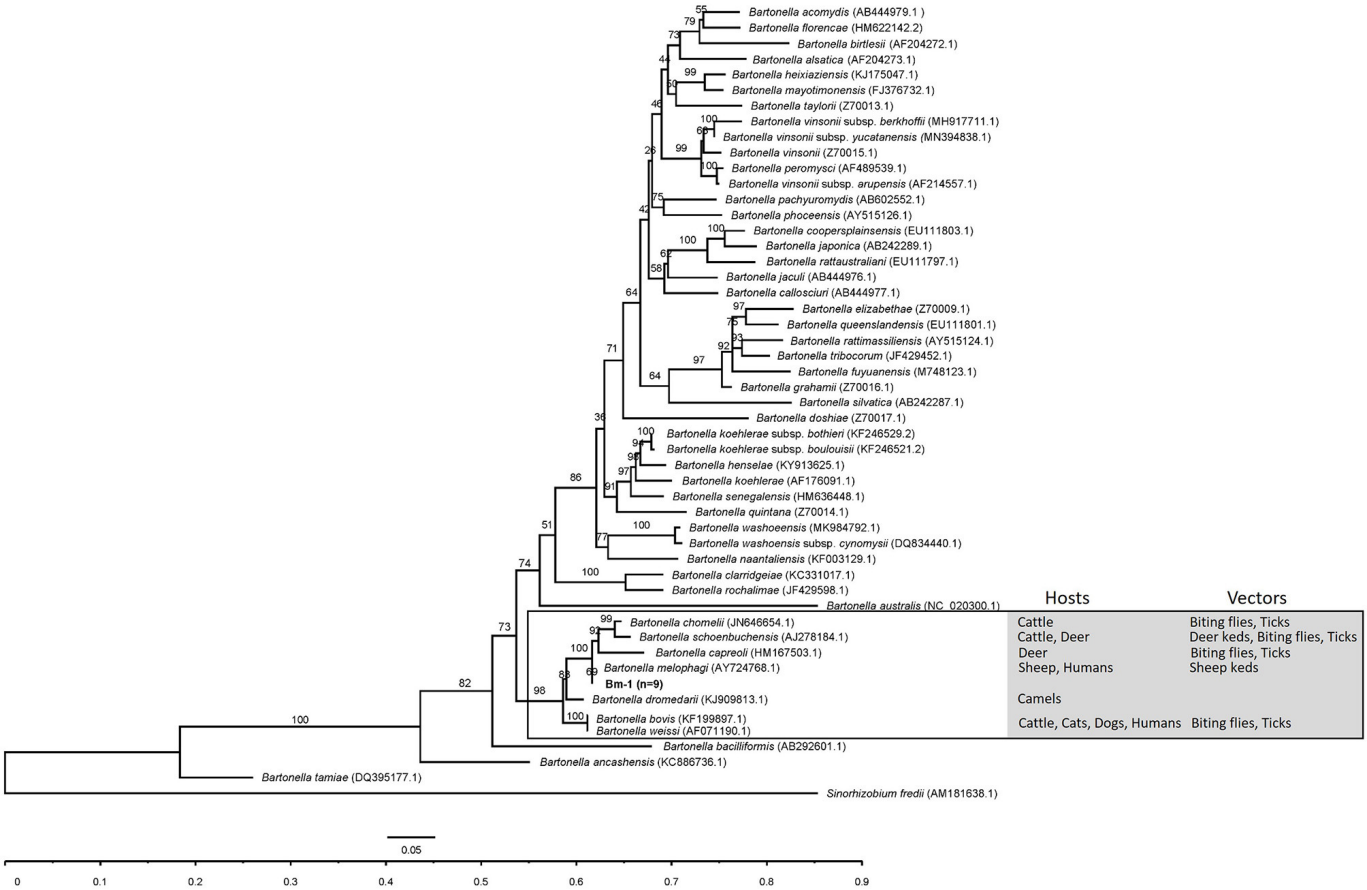
The phylogenetic tree of *Bartonella gltA* sequences was constructed by using maximum likelihood (ML) and GTR+I+G4+F as evolutionary models. The sequences detected in this study are shown in bold, and the numbers at the nodes correspond to bootstrap values. *Sinorhizobium fredii* was used as the out group.

**Figure 3:**
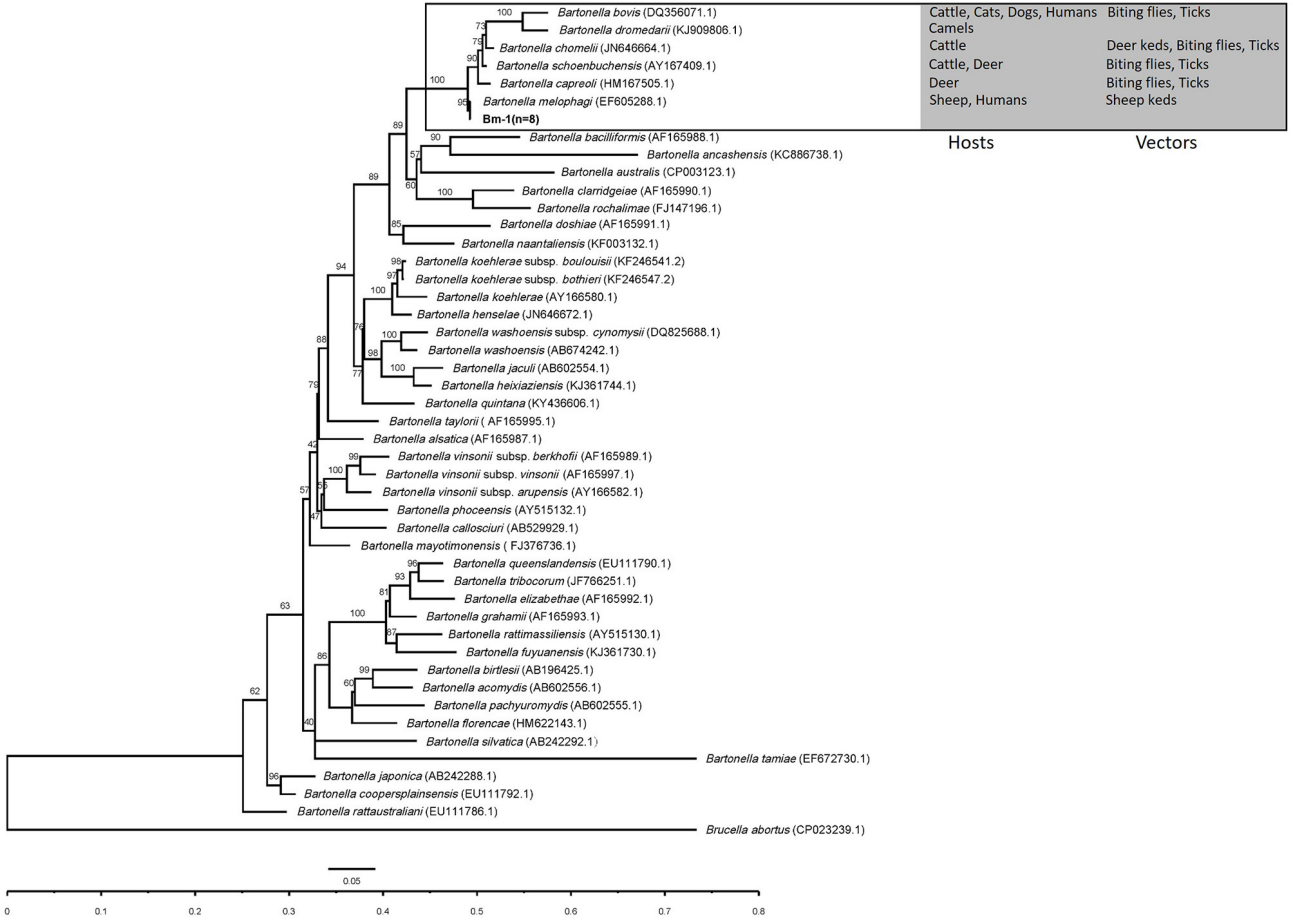
The phylogenetic tree of *Bartonella rpoB* sequences was constructed by using maximum likelihood (ML) and HKY+I+G4+F as evolutionary models. The sequences detected in this study are shown in bold, and the numbers at the nodes correspond to bootstrap values. *Brucella abortus* was used as the out group.

In the phylogenetic inference based on *gltA* and *rpoB* genes, the *gltA* and *rpoB* sequences obtained in this study were clustered into a branch of *Bartonella* associated with ruminants and supported by 98 and 100% bootstrap analysis. The *gltA* and *rpoB* sequences of *B. melophagi* in this study were closest to AY724768.1 and EF605288.1, respectively (Figures 2, 3).

## Discussion

Our study reports the prevalence of *Bartonella* in ticks in the border area of Xinjiang, China. A total of 1,549 tick samples were collected in the current study, and 2.19% (340/1,549) contained *Bartonella* DNA assayed by *ribC*-PCR. The *gltA, rpoB* and ITS genes of the *ribC* gene-positive samples were selectively amplified, and BLASTn analysis showed that the *gltA, rpoB*, ITS and *ribC* genes were all *B. melophagi*. This may be due to the potential pitfall in this study, because the positive control we selected is *Bartonella melophagi*. In view of this, more follow-up studies are needed, such as using another *Bartonella* as a positive control. Previous studies on *Bartonella* in ticks showed that the positive rates were 2.40% (7/292) in China (41), 2.48% (23/929) in 16 states of the USA (42), 2.32% (26/1,119) in California (43), and 2.2% (5/133) in Korea (15). Our results are similar to those reported previously. Another investigation of *Bartonella* in ticks from different countries reported positive rates of 1.05% (2/191) in Lithuania (44), 6.86% (19/277) in Algeria (9), 10.31% (13/126) in Brazil (12), 18.04% (57/316) in Poland (45), 10.4% (13/125) and 15.7% (40/254) in the Brazilian Cerrado and Taiwan (11, 12), 4.0% (8/200) in Malaysia (14), 5.8% (5/85) and 2.5% (1/40) in Korea (15), 8.56% (25/292) in North Korea (16), and 0.4% (1/234) in Pakistan (17). Previous studies on *Bartonella* in other ruminant-related blood-eating arthropods showed that the positive rates were 78.79% (26/33) in Hippoboscidae flies from Algeria (9) 4.21% (4/95) and 84.62% (11/13) in *H. tuberculatus* from the Brazilian Cerrado and *H. quadripertusus* from Israel (12, 13), 2.93% (16/546) in *C. felis* from Tunisia (18), 100% (133/133) and 36.87% (104/282) in *M. ovinus* from Central Europe and northeastern Algeria (19, 20), and 84.75% (50/59) and 93.75% (45/48) in *L. cervi* from Norway and Europe (6, 8). There were differences in the positive rates of *Bartonella* in ticks from different countries and regions, which may be related to the location, number, detection methods and ecological environment of the collected samples. Interestingly, the positive rate of *Bartonella* is also different in different arthropods. Because *Bartonella* species are mainly transmitted through vectors, it is speculated that the prevalence and richness of specific arthropods play a vital role.

Xinjiang is the largest province in China and is rich in tick resources. Forty-two species of ticks belonging to nine genera were reported in Xinjiang, accounting for more than 1/3 of the total number of tick species in China (26, 46, 47); the wide distribution of ticks has a significant impact on the development of animal husbandry and on public health. In recent years, *B. melophagi* was detected in *M. ovinus* from northeastern Algeria (20), China (31), central Europe (19), the western United States (48), and northern Oromia, Ethiopia (12). *B. melophagi* was detected in white-tailed deer (49) and human blood (50) in the United States. Xinjiang is located in the hinterland of Eurasia and has a land border of more than 5,600 km bordering Russia, Kazakhstan, Kyrgyzstan, Tajikistan, Pakistan, Mongolia, India, and Afghanistan. With the proposal of the Belt and Road Initiative of China, import and export trade becomes more frequent, and *Bartonella* may spread among different countries and regions with the import and export of trade products.

In the phylogenetic inference, the *gltA* and *rpoB* sequences obtained in this study were clustered into a branch with *Bartonella* related to ruminants. In the cluster, the hosts of *Bartonella* were mainly ruminants, such as cattle, sheep, camel, and deer, and the vectors of *Bartonella* mainly included deer keds, sheep keds, biting flies, and ticks (Figures 2, 3). However, ticks can be invoked as vectors for several ruminant-related *Bartonella*. This suggests that certain vectors, especially ticks, may play a key role in the spread of *Bartonella*. At the same time, ticks have a wide range of hosts, which makes it possible for *Bartonella* to spread across species through ticks. Ruminant-related *Bartonella* clustered into one branch, which may be explained by the relationship between *Bartonella* and the host. A host-specific association between *B. washoensis* and squirrel was revealed by using multisite sequence analysis. The results showed that *B. washoensis* may have co-speciated with the host squirrel. The phylogenetic relationships showed that the *B. washoensis* strain of *Spermophilus* is mainly related to the host genus rather than to its geographic origin (51). There may be a symbiotic relationship between *M. ovinus* and *B. melophagi* (8), and *B. melophagi* may transfer between *M. ovinus* and sheep (48). Interestingly, we also detected *B. melophagi* in ticks collected from cattle (Table 3). The coparasitism of ticks and *M. ovinus* in sheep may be considered a route of *B. melophagi* transmission. At present, there are no reports on associations between *B. melophagi* and sheep disease. *B. melophagi* was isolated from two women with a history of animal contact (50), which may suggest that *B. melophagi* is a possible human pathogen. In brief, our study provides evidence for the presence of *B. melophagi* DNA in ticks collected from cattle and sheep; however, further studies are needed to demonstrate that ticks are the vectors of *B. melophagi*. Additionally, we need to systematically investigate the prevalence of *Bartonella* in ticks, domestic mammals and people with a history of animal contact in Xinjiang, China. In general, this study is an important step in the control and public health safety of tick-borne *Bartonella* disease in mainland China and its neighboring countries.

## Conclusions

In this study, molecular analysis was used to assess the presence and genetic diversity of *B. melophagi* in ticks collected from sheep and cattle from Xinjiang, China. This study provides new information on the presence and identity of *B. melophagi* in ticks from sheep and cattle. Ticks are used as the transmission medium of *Bartonella*, and it is necessary to investigate the prevalence of *Bartonella* in ticks, animals and people with animal contact.

## Data Availability Statement

The datasets presented in this study can be found in online repositories. The names of the repository/repositories and accession number(s) can be found at: https://www.ncbi.nlm.nih.gov/genbank/, MT536936.1; https://www.ncbi.nlm.nih.gov/genbank/, MT549891.1; https://www.ncbi.nlm.nih.gov/genbank/, MT549892.1; https://www.ncbi.nlm.nih.gov/genbank/, MT549893.1; https://www.ncbi.nlm.nih.gov/genbank/, MT549894.1; https://www.ncbi.nlm.nih.gov/genbank/, MT549895.1; https://www.ncbi.nlm.nih.gov/genbank/, MT635398.1; https://www.ncbi.nlm.nih.gov/genbank/, MT534396.1.

## Author Contributions

JN and QR performed experiments. HL, MA, YLu, and ZM participated in sample collection. GL, ZC, JinL, and QR identification of tick samples. JN, JG, WL, XX, ZQ, ZW, and YT performed data analysis. JW, YLi, GG, JiaL, HY, and GL revised the manuscript. All authors read and approved the final manuscript.

## Conflict of Interest

The authors declare that the research was conducted in the absence of any commercial or financial relationships that could be construed as a potential conflict of interest.
